# Experiences of Bereaved Family Members Receiving Commemorative Paintings: A Qualitative Study

**DOI:** 10.1001/jamanetworkopen.2020.27259

**Published:** 2020-12-21

**Authors:** Marisa A. Azad, Marilyn Swinton, France J. Clarke, Alyson Takaoka, Meredith Vanstone, Anne Woods, Anne Boyle, Neala Hoad, Feli Toledo, Joshua Piticaru, Deborah J. Cook

**Affiliations:** 1 Division of Infectious Diseases, Department of Medicine, University of Ottawa, Ottawa, Ontario, Canada; 2 Department of Health Research Methods, Evidence and Impact, McMaster University, Hamilton, Ontario, Canada; 3 Division of Palliative Care, Department of Family Medicine, McMaster University, Hamilton, Ontario, Canada; 4 Department of Family Medicine, Department of Medicine, St Joseph’s Healthcare Hamilton, Hamilton, Ontario, Canada; 5 Critical Care Research, St Joseph’s Healthcare Hamilton, Hamilton, Ontario, Canada; 6 Department of Spiritual Care, St Joseph’s Healthcare Hamilton, Hamilton, Ontario, Canada; 7 Department of Medicine, McMaster University, Hamilton, Ontario, Canada

## Abstract

**Importance:**

Although family members of patients who die in the intensive care unit commonly experience long-term psychological distress, end-of-life bereavement support programs for such relatives are uncommon. Whether art influences the grief experience of families is largely unexplored.

**Objective:**

To explore the influence of personalized paintings created to honor deceased critically ill patients on family members’ bereavement experience.

**Design, Setting, and Participants:**

A qualitative descriptive analysis was conducted of semistructured interviews of grieving relatives who received a painting after the death of their loved one. The deceased patients were from a 21-bed medical-surgical intensive care unit. Eleven families were invited to receive a painting, of whom 1 family declined. A total of 22 family members of 10 patients who died in the intensive care unit were interviewed in the study between July 11, 2017, and May 19, 2019.

**Interventions:**

Patients were enrolled in an end-of-life care program that elicits and implements wishes of patients and their families to bring peace during the dying process. Selected families of 10 decedents were invited to receive a painting to honor their loved one 1 to 10 months after the patient’s death. Using details about the patient’s life story, the artist created individualized paintings to commemorate each patient.

**Main Outcomes and Measures:**

The experiences of family members receiving a personalized painting and its reported influence on their grieving experience.

**Results:**

The family members of 10 decedents (mean [SD] age, 60 [14] years; 5 women [50%]; 8 White patients [80%]) were interviewed. The central theme of art to facilitate healing was illustrated through the following domains: the cocreation process, painting narratives, postmortem connections, and legacy. The process of cocreating the paintings with the artist and family members involved reminiscing, storytelling, and creativity. Family members emphasized the role of art to facilitate healing, exemplified through connections with images portrayed that deeply resonated with memories of their loved one. Participants indicated that the paintings validated that the patient was remembered, helped families feel less alone during a time of grief, honored the loved one’s life, and enhanced connections between family members and clinicians.

**Conclusions and Relevance:**

This qualitative study’s findings suggest that the creation of personalized paintings commemorating the lives of patients may help foster legacy and postmortem connections with clinicians and may help family members in their healing process.

## Key Points

**Question:**

What potential role do personalized paintings that are created to honor the lives of deceased patients in the intensive care unit have for grieving family members, and how might they influence family members’ experiences with bereavement?

**Findings:**

In this qualitative study, families described personalized paintings as representing a legacy of their loved one. Participants indicated that the paintings were symbolic of the care their loved one received, validated the sentiment that the patient was remembered, and helped the families feel less alone during their time of grief.

**Meaning:**

The creation of personalized patient paintings may foster postmortem connections between family members and clinicians and may ease bereavement following a death in the intensive care unit setting.

## Teaser

This qualitative study uses semistructured interviews to explore the influence of personalized paintings of patients who died in the intensive care unit on family members’ bereavement experience and grief process.

## Introduction

Despite advances in life-sustaining measures, mortality in the intensive care unit (ICU) may be as high as 30% to 50%.^1,2^ With such an emphasis on devices to monitor, diagnose, and treat critical illness, family members of dying or deceased critically ill patients often suffer from long-term psychological distress due to repeated exposures to traumatic events, such as mechanical intubation and invasive interventions.^3^ Although consensus groups recommend bereavement support for families,^4,5^ most ICUs do not offer such programs.^6^ The 3 Wishes Project (3WP) helps to bring peace to the final days of a patient’s life and comfort families.^7,8^ The project involves eliciting wishes from the patients, family members, and clinicians in an effort to honor the dying patient and comfort family members. Wishes can range from playing favorite music to renewal of wedding vows to word clouds, a visual representation of words collated by families to represent aspects of the patient’s life before critical illness. Research suggests that making a word cloud to remember someone after their death reaffirms their life.^9^

Art is a universal language that connects people in ways words cannot, and it may positively influence the grief experience. The inherently personal and emotional nature of art makes it a powerful tool of expression that can create a connection between subject matter, viewers, and artist.^10^ Visual arts–based interventions have been implemented for patients with cancer, trauma, and other chronic illnesses, demonstrating the potential to improve psychological well-being and even medical outcomes for those engaged in the artistic process.^11^ Art has also been identified as an important educational tool to foster empathy and decision-making in medical trainees.^12,13,14^ The objective of this study was to explore the influence of personalized paintings, made in honor of deceased critically ill patients, on grieving family members.

## Methods

### Study Design

This study was conducted in a 21-bed medical-surgical, university-affiliated tertiary ICU from July 11, 2017, to August 31, 2020. We obtained ethics approval from the Hamilton Integrated Research Ethics Board. Framed with a pragmatist philosophical approach,^15^ this study used qualitative description,^16,17^ documented per reporting guidelines for qualitative research.^18^ This study followed the Consolidated Criteria for Reporting Qualitative Research (COREQ) reporting guideline.

### Study Participants

This study was nested within the 3WP, a palliative care program in place for 5 years at the time of this study. Critically ill patients were enrolled due to a poor prognosis or withdrawal of life support. Family members were purposively sampled and invited to receive a personalized painting based on existing relationships with the 3WP staff. Family members provided written, informed consent for participation and for the publication of their responses. The 3WP coordinator approached family members in person or by phone 1 to 10 months after the patient’s death to determine whether the gift of a painting would be welcome. Family contact was spaced at time intervals to ensure that the artist had sufficient time to complete each painting. The artist (M.A.A.) was an internal medicine resident with 15 years of painting experience and several pieces displayed in galleries across Canada.

### Paintings

The artist created paintings following conversations with family members and through preestablished relationships forged between staff and family members in the 3WP. Examples of information elicited by the artist included what mattered most to the patient, their goals, dreams, and defining life moments (eAppendix 1 in the Supplement). Paintings incorporated imagery and stories reflecting the patients and were further informed by reviewing word clouds.^9^ Beyond representing images that symbolized the identity of the patient, the artist incorporated the theme of hope in each painting, using visual vocabulary specific to the individual’s life. Paintings were created using an oil medium, and multiple prints were gifted to families, if requested, using an art tablet. The artist presented the paintings to families at the hospital upon completion and explained the artistic process and meaning behind each painting.

### Interviews

After the painting presentations, we conducted 30- to 40-minute semistructured interviews with 22 family members of 10 deceased patients enrolled in the 3WP who received a personalized painting. Interviews were framed with open-ended questions (eAppendix 2 in the Supplement) and conducted by a qualitative researcher (M.S.). Painting presentations and interviews were in person for all but 1 family; the final presentation and interview were conducted via videoconference due to artist relocation and coronavirus disease 2019 pandemic precautions.

### Analysis

Interviews were digitally recorded, transcribed, and anonymized. We identified themes through conventional content analysis.^19^ Three investigators (M.A.A., M.S., and A.T.) independently completed line-by-line open coding on 3 transcripts and then developed a preliminary list of codes through consensus. The lead analyst (M.S.) coded the remaining transcripts. Three investigators (M.S., A.T., and D.J.C.) reviewed coding reports, assessed and confirmed data saturation,^20^ and organized the codes into meaningful categories. Higher-level clusters of categories were created based on the relationships between the code and categories.^21^ A study audit trail was maintained to document all decisions related to coding and analysis.^22^ NVivo, version 11 (QSR International) was used for data management.

The research team reflected a group of interdisciplinary clinicians and nonclinician qualitative researchers. Clinicians represented medicine, nursing, respiratory therapy, and spiritual care, and all the clinicians cared for dying patients in the ICU. The research team had no relationship with participants before their arrival to the ICU, where some of us formed professional relationships based on our clinical or research roles before offering the family a commemorative painting.

## Results

The family members of a total of 10 decedents (mean [SD] age, 60 [14] years; 5 women [50%]; 8 White patients [80%]) were included in this study. Patient characteristics are summarized in Table 1. Of the 11 families invited to receive a painting, 1 family declined, describing the patient as a very private person. Table 2 summarizes the 22 family members who received a painting, all of whom agreed to be interviewed. The themes and imagery represented in each painting are shown in Table 3. Nine paintings are displayed in the Figure and in eAppendix 3 in the Supplement. One painting is accessible in a previous publication.^23^

**Table 1.** Patient Characteristics

Characteristic	No. (%)^a^
Age, mean (SD), y	60 (14)
Women	5 (50)
Race/ethnicity	
Aboriginal	1 (10)
Black	1 (10)
White	8 (80)
APACHE II score, mean (SD)	31 (8)
ICU admitting diagnostic category	
Respiratory	8 (80)
Gastrointestinal	1 (10)
Cardiac	1 (10)
Spiritual belief	
Christian	8 (80)
Protestant	3 (30)
Catholic	2 (20)
Denomination not specified	3 (30)
None indicated	2 (20)
Time in ICU, median (IQR), d	24 (0-127)
Time from ICU admission to enrollment in 3WP, median (IQR), d	21 (0-127)
Time from 3WP enrollment to death, median (IQR), d	3 (0-4)
3WP initiated by	
3WP team PI	5 (50)
Resident	1 (10)
Nurse	2 (20)
Spiritual care	1 (10)
Other	1 (10)
Reason for enrollment in 3WP	
Poor prognosis	8 (80)
Withdrawal of life support	2 (20)
Advanced life support	
Mechanical ventilation	10 (100)
Vasopressors, inotropes	4 (40)
Hemodialysis	3 (30)

Abbreviations: 3WP, 3 Wishes Project; ICU, intensive care unit; IQR, interquartile range; PI, principal investigator.

^a^ Values expressed as No. (%) unless otherwise specified.

**Table 2.** Family Member Characteristics

Painting No.	No. of family members interviewed	Roles of family members interviewed	Months between patient’s death and family interview
1	5	Mother, father, sister, boyfriend, grandmother	1.7
2	2	Wife, son	1.2
3	1	Wife	2.4
4	2	Husband, daughter	4.6
5	2	Sister, niece	4.7
6	2	Husband, daughter	6.7
7	1	Sister	8.3
8	1	Husband	9.6
9	4	Mother, father, sister, brother	10
10	2	Daughter, son-in-law	16.5^a^

^a^ Delay due to artist relocation.

**Table 3.** Painting Characteristics

Painting	Title	Imagery	Major themes, experiences, and stories
1	*Tooth of the Lion*	Hummingbird, French wine glass, dandelions, red lupins	Creativity, a mother’s love, the healing power of dandelions, spirituality, love of one’s heritage, family
2	*What the Earth Showed Me*	Vegetable garden, wristwatch, soil, plant with exposed roots, wedding band	Digging up the past, gardening, hope, the story of our marriage, a quiet strength, family
3	*This Land*	Australian landscape, sunset, carved heart in a tree with the patient and partner’s initials	Companionship, exploration, our next great adventure, peace, belonging
4	*For Eartha*	White dove, book of traditional hymns, the language of flowers, *Through the Looking Glass* by L. Carroll	Spirituality, love of God, love of family, honesty, kindness, to be reborn
5	*Sheba*	Sheba the cat (family pet), blue jay, snow, daffodil in full bloom	Sheba as a family member, circle of life, resilience
6	*The Breath*	White daisy with water droplets, orange goldfish	“It was her decision to be taken off the ventilator,” sisterhood, family, independence, sunsets, calmness
7	*Dear Michelle*	Candlelit scene, letter to one's mother, forget-me-nots, hidden smile in the vase of flowers, tears of happiness	Happiness, family, strength, legacy, the power of a smile
8	*For Rose*	A chickadee clutching a rose	“She will never leave my side,” everlasting love and friendship, personal growth, serenity
9	*The Fisherman*	Kingfisher facing a rainbow trout, swirling background which represents a lake	“Loved being at the lake”, the drama of fishing, patience, togetherness, never giving up
10	*Looking Back*	Bird in flight, unraveling yarn, the sky	Genealogy, family, healing, photography, peace

**Figure.**
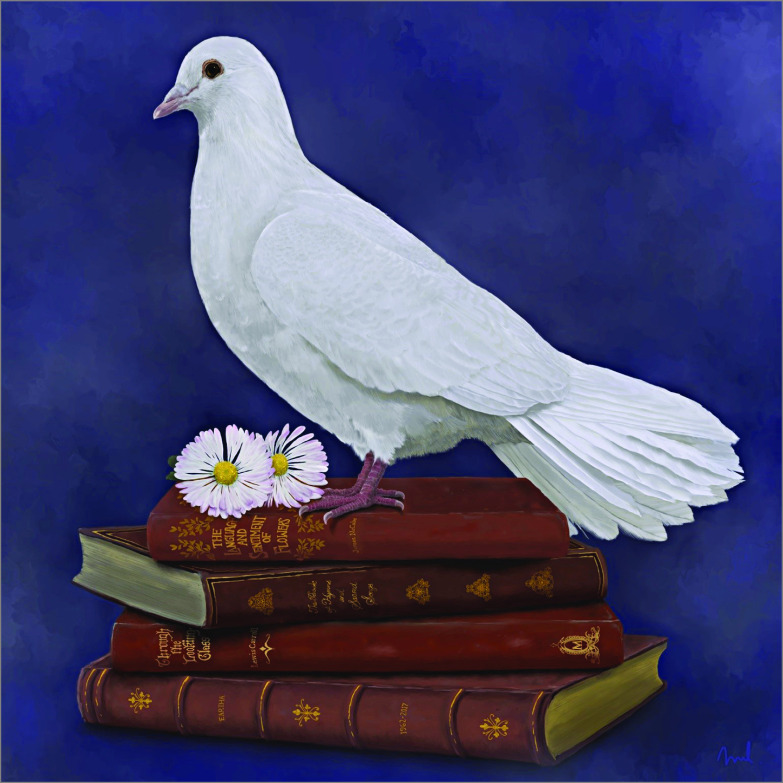
Painting No. 4: *For Eartha* An example of one painting created in this study by the artist.

### Art to Facilitate Healing

The central theme of art to facilitate healing is illustrated through the following domains, labeled as subheadings below: the cocreation process, painting narratives, postmortem connections, and legacy. The process of cocreating the paintings with the artist and family members involved reminiscing, storytelling, and creativity. Seeing the final painting evoked strong emotional responses from family members, prompting them to share their own interpretations of the imagery and symbols, describing how the paintings captured their loved one. This resulted in close connections for family members with the artist and the 3WP team. The painting was a tangible keepsake, created in honor of the deceased.

#### The Cocreation Process

The conceptualization of each painting involved family member input, facilitated through postmortem telephone communication with the artist and the research team. Conversations elicited patient values, passions, goals, and memorable life events cherished by family members. This dynamic process fostered a sense of connectedness through sharing ideas and listening to stories: “…[the artist] even told us about the dandelion, and I’m thinking, oh my gosh, we were just talking about the dandelion…I mean, he was even eating the leaves in his salad, you know?” (mother, painting 1).

Some family members were overwhelmed by the invitation to participate in such a project and expressed not knowing what to expect. “So I told [the 3WP coordinator] about the birds and about the cat and then she called [artist] because I wasn’t sure if I wanted to have her do it or not, how I would feel, and so she talked to [artist] and [artist] talked to me, and we said yes, we would like that” (sister, painting 5).

Once the paintings were complete, family members recognized how their own experiences of their loved one were integrated with the artist’s interpretation, enabling them to see their loved one in a new light. One family member articulated,

I think it’s interesting to have someone pick up on stories, because I never got to meet [the artist] until after the fact and so I talked to her on the phone for about an hour, but [the 3WP project coordinator] had told her already a lot of things…[the artist] put things in there…one thing was expected and one thing was unexpected (sister, painting 6).

Previous relationships built through the 3WP provided a strong foundation on which to engage family members in the cocreation process. One family member described:

They had a relationship with us during our 2 weeks here, and that relationship was more important than anything…a relationship is all about, not just speaking, right, but listening, and I think that that’s what they did, because they certainly listened well and captured everything, and they listened to our stories and they listened to us talk about [patient], and they captured everything (husband, painting 7).

#### Painting Narratives

Images in the paintings strongly resonated with family members and were tied to powerful narratives that prompted family members to share stories when first viewing the painting:

Well, I just feel that that’s [wife’s name]…sitting there, looking at me. And she’s got her favorite flower, a rose. She loved the roses. And it didn’t matter what the color was, as long as it was a rose. And she used to get mad when she went into the stores and she picked them and smelled them and there was no smell to them (husband, painting 8).

Family members felt that the images uniquely captured many dimensions of their loved one’s life story. Referencing images of a vegetable garden, wristwatch, soil, and a wedding band, one son reflected, “It was a perfect representation of my dad…I mean, you look at it and you know, everything’s in there” (son, painting 2). Families drew both concrete and abstract connections from images, relating them to qualities of their loved one. “[The] salmon going upstream, fighting against everything, that symbolizes who he is and who he was as a person…” (brother, painting 9). One family member described the books in the painting: “I imagine it as her life story…” (husband, painting 4). Families felt touched by the carefully selected objects portrayed through each painting, expressing how the symbolism was personal. “I think the biggest thing for me is the letter that [daughter] wrote to her mother and to have that right in the painting is, it’s so personal, and it’s us” (husband, painting 7).

#### Postmortem Connection

This project fostered strong connections between family members, the artist, the 3WP team, and the clinicians. Many families emphasized “the connection afterwards” (mother, painting 9), with staff as the most meaningful part of their experience. Family members spoke to the importance of being cared for after leaving the hospital, describing being called about the painting and the value of connecting to staff: “For me, it’s helped me a lot with the grieving part. Like the [word cloud]; talking to [artist] about it, things like that, you know? Yeah, the care and the aftercare and [the 3WP coordinator] and the team…incredible people” (sister, painting 9).

Hope for a continued relationship beyond the end of the project was expressed in some cases:

And [the program] has really carried on beyond our time here for those 2 weeks, and that’s what is so meaningful…we’re more engaged in the program now because of the relationship they’ve continued with us afterward, so I’m hopeful that this isn’t the end after the beautiful painting, that we can keep that relationship going and we can be involved in the program however we can to make it lasting (daughter, painting 7).

#### Legacy

Families described paintings as something that could be passed down in the family and as a way in which their loved one could live on: “It’s the essence of my father…he’s not gone. He’s with us and he’s within that painting” (son, painting 2). Family members described using the painting as a catalyst for reminiscing: “…one’s memory of things as you age diminishes. But the painting will always be a fresh reminder of his life...” (wife, painting 3). Most family members described where they planned to hang the painting. For example, one family member planned to place his painting in the kitchen where he could speak to his wife each day. Families acknowledged how the painting would help to convey who the patient was to future family members:

…[T]here’s no end to the life span of the painting, my grandkids will be part of what she was…it’s so important to us that we continue her legacy, for the people that are going to become part of our family that are not part of our family yet, but for them to know her…my daughter and I are who we are because of her (husband, painting 9).

Paintings validated that their loved one was not forgotten: “I am humbled and touched that they thought that [the patient’s] life was important enough to be remembered in this way” (wife, painting 3). One family member described how the initiative and effort of the artist to honor her loved one was comforting: “It gave me peace that he was actually honored this way, and it gave me peace that someone from this ICU really, really wanted to know him” (wife, painting 1). Paintings were symbolic of the care and compassion that patients and families experienced: “It tells me that somebody else cared about [the patient] and somebody else cared about me…cares about the 2 of us. And that’s beautiful” (husband, painting 9). Another family member shared,

So I’ll have it hanging there with all her little things that make me think of her and I’ll think about that fish and I’ll think about that daisy and I’ll think about the people that cared enough to remember her story about her passing so that she wasn’t just forgotten (sister, painting 6).

We identified several forms of grief work, manifest as additional postmortem connections.^24^ This work included relatives’ initiatives to film a documentary about the project, speak at a conference to share their experience with the project, write appreciation letters to the hospital leadership, and encourage an art exhibition of these paintings.

## Discussion

In this qualitative study, we evaluated the influence of personalized paintings on family member grief experiences. Families described conceptualizing and receiving personalized paintings as healing, helping to bring them peace and feel cared for. The cocreation of paintings facilitated the sharing of powerful stories reflecting who the patient was, validating how family members remembered them. Meeting to receive the paintings fostered meaningful postmortem connections between family members, the artist, and research staff. For families, the paintings contributed to the legacy of the deceased.

Art therapy during bereavement has been associated with meaning-making through continuing bonds between the deceased patient’s family and the patient.^25^ Personalized paintings created within this project differ from traditional forms of art therapy wherein art, created by clients and facilitated by the certified art therapist, is used to promote mental health and well-being.^26^ In this project, art was coconceptualized with the family, painted by the artist, and gifted to the family. Presentation of these paintings to families prompted commentary and reflection on concrete and abstract connections between shapes, colors, and compositions within the paintings. Family members stated that paintings symbolically captured aspects of their loved one’s life and also reflected the memories of others. This representation led families to discover new ways of remembering patients, resulting in further cultivation of positive memories.

We posit that the meaning experienced by the families may in part be related to the time a clinician took to get to know their loved one rather than being wholly dependent on a specific skill (ie, painting). Individual clinicians may have unique ways to demonstrate their care in a tangible way (eg, knitting, baking, or playing music). In the 3WP,^9,27^ we have observed the opportunity for postmortem connections through the cocreation process of other art forms, such as keepsakes. This process echoes a finding of Riegel and colleagues,^28^ who described the value of memory-making activities at the end of life that rely on a cocreation process and are not dependent on the skill of an artist. Furthermore, emerging evidence shows the potential value of art therapists cocreating artwork with patients who may not be able to physically participate owing to illness; there may be other opportunities to forge connections and memories at the end of life using this technique.^28^

An area of this study that warrants further exploration is that of different cultures’ understanding and perception of art. We found all family members, regardless of cultural or socioeconomic background, to be open to receiving the gift of a painting and forthcoming during the cocreation process. Regardless of whether family members had a background in the arts, participation in storytelling during painting presentation occurred spontaneously. Many family members presented some of their own artistic creations to the artist during the painting cocreation process. For example, one family shared with the artist many of their loved one’s sculptures, highlighting themes related to their Indigenous culture; in doing so, the artist was able to incorporate some of these themes into their painting. This family was so touched by this experience that they invited the artist to their loved one’s funeral and have remained in contact with her. Although art in its many forms has been and continues to be used by different cultures as a powerful form of communication,^29,30^ cultures and backgrounds do shape our understanding and perception of art.^31^

Meaning-making activities may help to reconstruct perceptions of interpersonal relationships in response to grief, thereby helping bereaved individuals to generate new world views, alleviating distress after a loss.^32^ Because the “meaning of one’s thoughts during grief may be contingent on their having meaning for someone else,”^33^^(p208)^ images of the patient’s life as seen through the eyes of the artist and informed through conversations with family members and clinicians yielded a tangible validation of memories, enhancing meaning during the bereavement period.

The creation of each painting was a dynamic process that in itself may have been beneficial for bereaved family members. Social support is a recognized predictor of positive psychosocial outcomes after a death.^34,35,36^ Family members have reported that a continued connection with clinicians after the death of a loved one is both desired^37,38^ and important for the grieving process.^39^ Shared reminiscence during bereavement has been a longstanding practice in grief work.^33^ Through sharing stories, bereaved family members can disclose emotions, engage in cognitive processing, and create social connections.^40,41^ In this study, the painting cocreation process resulted in stories shared and connections strengthened among the artist, relatives, and the research team. These interactions were a manifestation of postmortem care, helping family members feel less alone in their grief.

### Limitations

Limitations of this study include the small number of patients represented. We included family members with whom the bedside staff and the 3WP team had a relationship and for whom such a gift would be welcome. This purposeful sampling may have generated favorably biased interview responses, as this study was nested within a palliative care program involving eliciting and implementing personalized wishes at the end of life. We acknowledge that bereavement interventions have inherent risk: their effectiveness is dependent on the individuals involved, the degree of receptiveness,^42^ and the nature of the relationship with those providing the intervention.^43,44,45^ An invitation to receive the gift of a personalized painting could be viewed as an imposition. Strong relationships formed in the context of an established end-of-life program^7,8^ were foundational to the invitation. We avoided communicating that a positive response was expected, and we provided the family with ample opportunity to decline, as demonstrated by one relative who indicated that the patient would have been too humble to accept a painting created in his honor. We did not use quantitative instruments to explore the prevalence of grief symptoms; instead, our semistructured interviews were designed to ask neutral, open-ended questions to elicit reflections on the paintings honoring deceased patients and explore family member experiences. In addition, the transferability of this project is dependent on the availability and commitment of such an artist to take on a project of this magnitude, including the necessary resources. The creation of different types of personalized artwork for dying patients is worthy of further evaluation.

## Conclusions

In this qualitative study, we explored family member experiences of receiving personalized paintings in honor of the lives of deceased critically ill patients. Results suggest that artwork can support family members in grief and strengthen postmortem connections. Whereas humanity can be overshadowed by technology in the ICU, this study suggests that art can be used as a tool to engage patients and family members by encouraging clinicians to care with creativity and compassion and to assist family members with healing during the bereavement process.

## Notes

Supplement. **eAppendix 1.** Artist’s Initial Interview Template With Family **eAppendix 2.** Family Interview Template About the Art as Embedded in the 3 Wishes Project **eAppendix 3.** Paintings Created by the Artist for This Study
