# Supplementary material for: Experiences of Bereaved Family Members Receiving Commemorative Paintings: A Qualitative Study
Source: JAMA Netw Open. 2020 Dec 21;3(12):e2027259. doi: 10.1001/jamanetworkopen.2020.27259 (PMC7753900; doi:10.1001/jamanetworkopen.2020.27259)
Supplement: Supplement. — eAppendix 1. Artist’s Initial Interview Template With Family eAppendix 2. Family Interview Template About the Art as Embedded in the 3 Wishes Project eAppendix 3. Paintings Created by the Artist for This Study [file jamanetwopen-e2027259-s001.pdf]

Supplementary Online Content

Azad MA, Swinton M, Clarke FJ, et al. Experiences of bereaved family members receiving commemorative paintings: a qualitative study. *JAMA Netw Open*. 2020;3(12):e2027259. doi:10.1001/jamanetworkopen.2020.27259

eAppendix 1. Artist's Initial Interview Template With Family

eAppendix 2. Family Interview Template About the Art as Embedded in the 3 Wishes Project

eAppendix 3. Paintings Created by the Artist for This Study

This supplementary material has been provided by the authors to give readers additional information about their work.

© 2020 Azad MA et al. *JAMA Network Open.*

eAppendix 1. Artist's Initial Interview Template With Family

1. What mattered most to [name]?
2. What were [name's] passions, goals, and dreams in life?
3. What were some of [name's] proudest and most defining moments?
4. What memories has [name] left you with?
5. How would [name] have wanted to be remembered?
6. Is there anything else you would like to share with me about [name]?

© 2020 Azad MA et al. *JAMA Network Open.*

eAppendix2. Family Interview Template About the Art as Embedded in the 3 Wishes Project

Context for Painting Presentation:

The family member will already have been very familiar with the 3 Wishes Project. We will have previously explained that the painting would be a gift - a wish of the painter who is a clinician, and thus have obtained verbal consent for this wish. We will present the painting to the family members. The artist, Dr. Azad, will explain why the image was chosen and how it was personalized to reflect the patient. We will explain that the painting is an expression of gratitude on behalf of the artist to honour the patient. Alternatively, if the artist looked after the patient, we will explain that it is an expression of gratitude for the privilege of caring for the patient, and being allowed into their lives.

After the Painting Presentation and Informed Consent, Interview Guide:

Thank you for coming to meet with us today. I know it must be a very difficult time for you with your [role of patient] recently passing away. It means a lot to us that you came back to talk with us today.

With the 3 Wishes Project, we are trying to improve care for patients and families at end of life. Your feedback will help us. As I ask you some questions, please say whatever comes to your mind and describe your thoughts and feelings in as much detail as you can. Feel free to take your time answering. If there are any questions you don't want to answer please just let me know. We can take a break or stop at any time. Is it ok to start?

1) I understand there were a couple of things that were done for [patient's name] during the time when she was in hospital. Can you start out by telling me about the different wishes, or different things that were done for you and for [patient's name] during your time in the ICU? [probes: ask about specific wishes if the family member doesn't remember them; ask for more detail about the wishes as they talk about them].

2) One of the wishes was the Word Cloud. Can you tell me about your experience with the Word Cloud? Do you remember when the idea was first mentioned to you? If yes, do you remember how you felt when you first heard about it? If the family member was involved in developing the list of words for the Word Cloud: How did you come up with the list of words for the word cloud? How did it feel to help with those words?

3) Next, I'd like to ask you some questions about the painting you just received. How did you feel when you first saw the painting? Can you tell me a bit about the painting? Is there anything in the painting that stands out or resonates for you? Can you tell me how it feels to receive a gift like this? What does it mean for you to have this painting? Can you share how you felt when you first heard about the idea of the painting? [probes: did the artist phone you?] How was the timing of being approached about the painting? [probes: reasonable/too soon after your [patient's role]'s death?]

4) Has having the [name the wishes] and the painting had any influence for you, personally, on your experience of losing your [patient's role]? [probes: contact with the artist, contact with the 3WP project manager, bereavement experience].

5) Is there anything that you can think of that the 3 Wishes Project could have done differently? Is there something the project could have done that they didn't do?

6) Before we close off, I just wanted to know if you had anything more to share about your experience with the wishes, including the painting?

© 2020 Azad MA et al. *JAMA Network Open.*

eAppendix 3. Paintings Created by the Artist for This Study

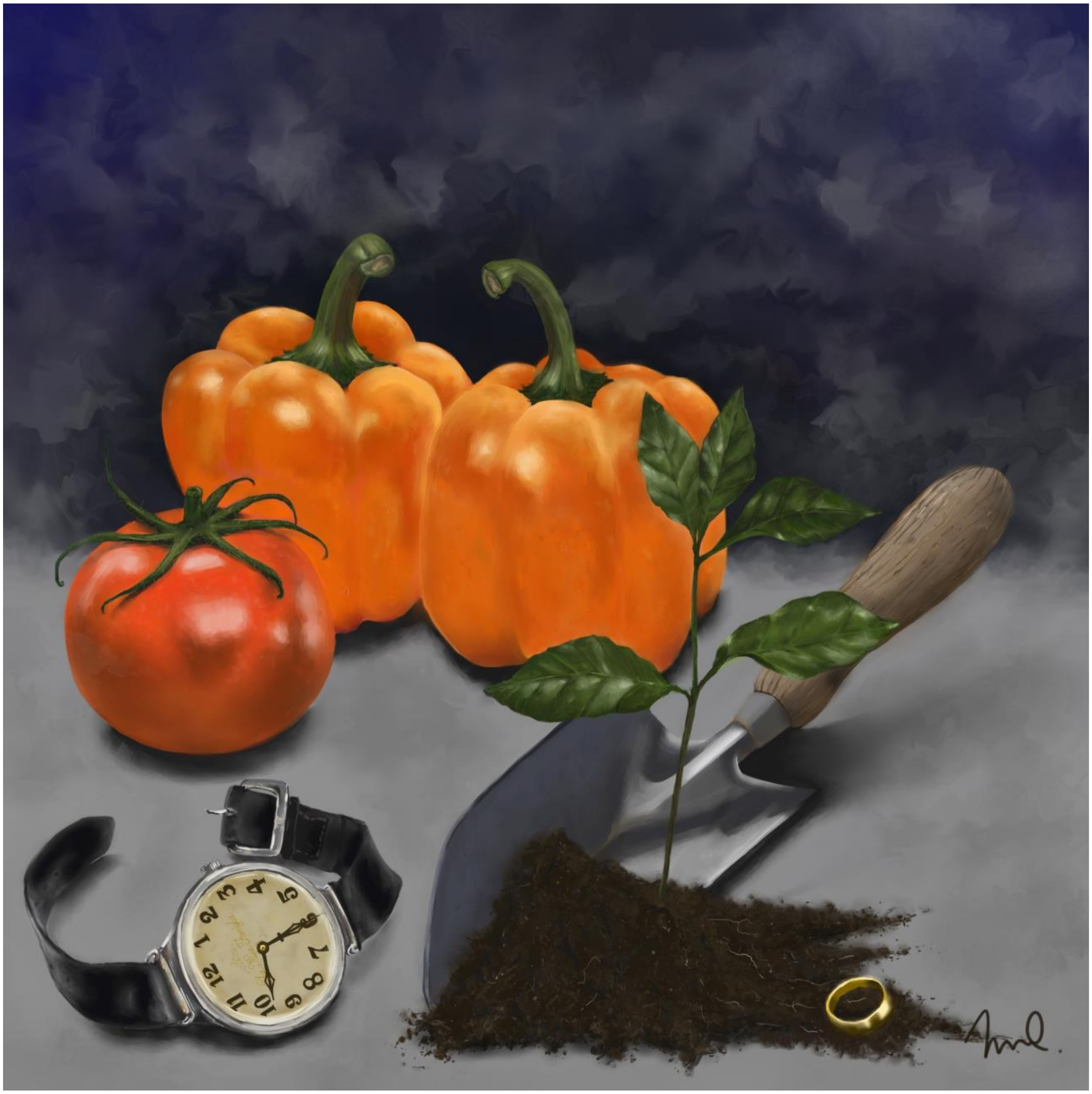

Painting 2: *What the Earth Showed Me*

© 2020 Azad MA et al. *JAMA Network Open.*

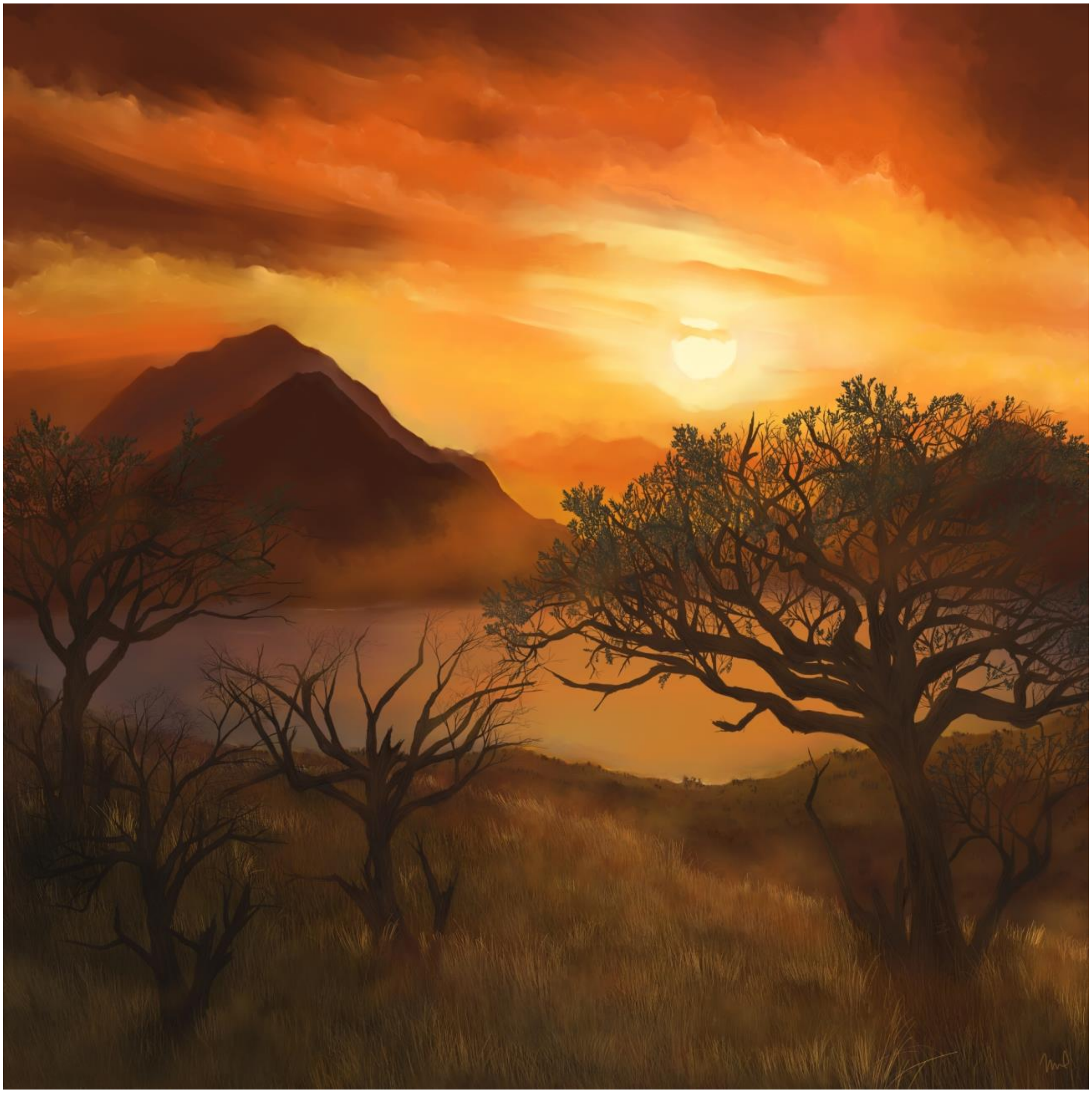
Painting 3: *This Land*

© 2020 Azad MA et al. *JAMA Network Open.*

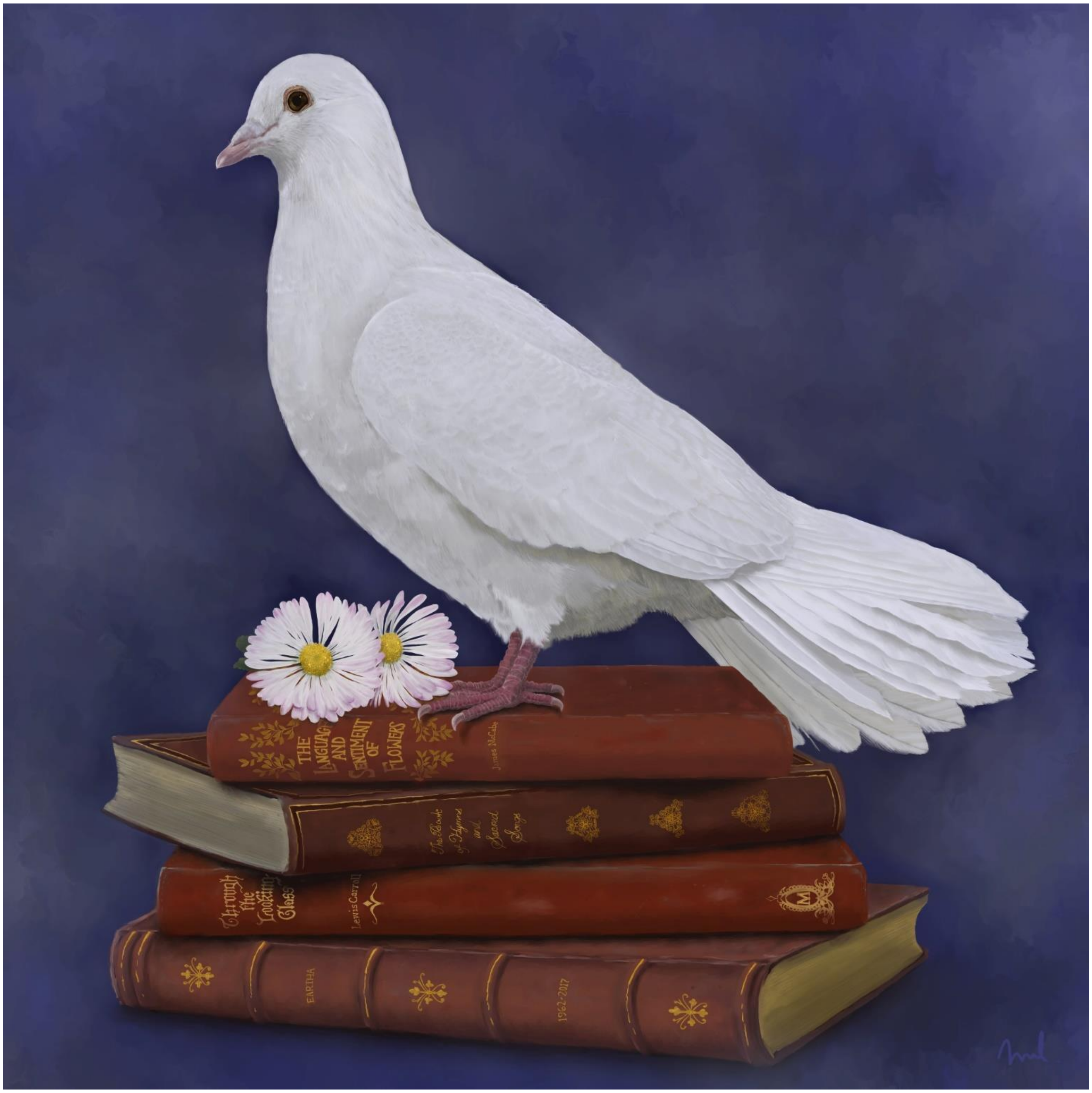

Painting 4: *For Eartha*

© 2020 Azad MA et al. *JAMA Network Open.*

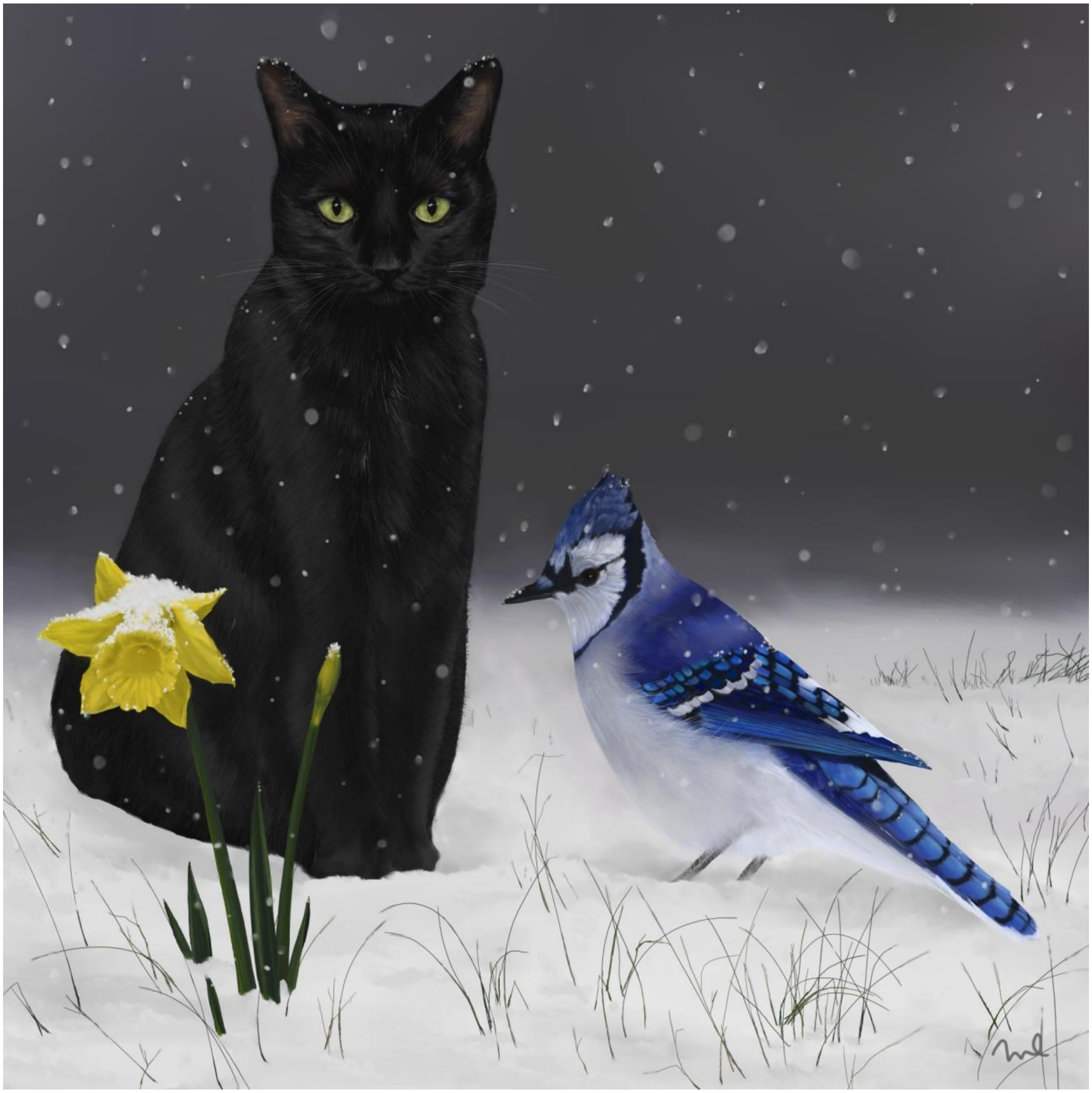
Painting 5: *Sheba*

© 2020 Azad MA et al. *JAMA Network Open.*

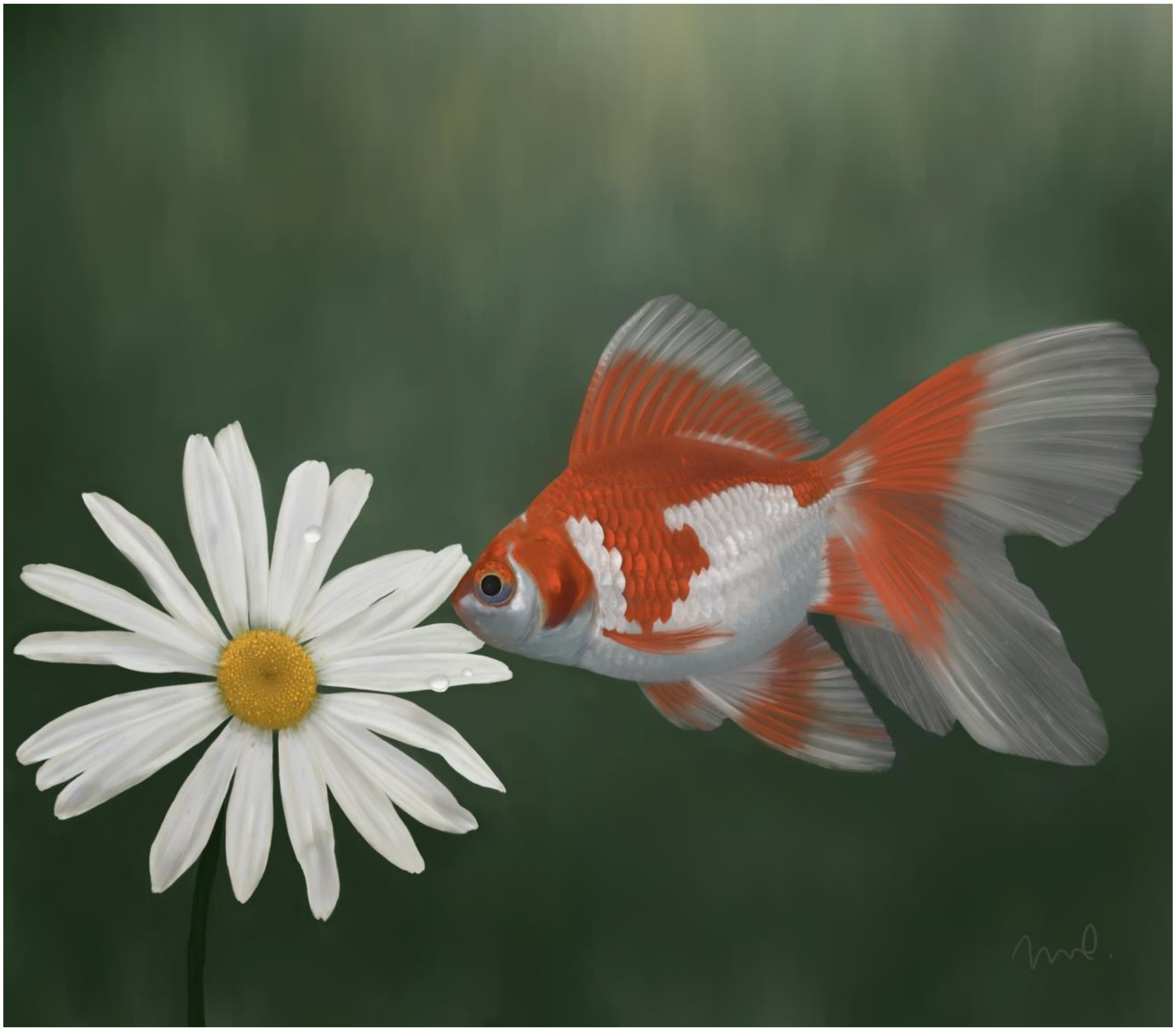

Painting 6: *The Breath*

© 2020 Azad MA et al. *JAMA Network Open.*

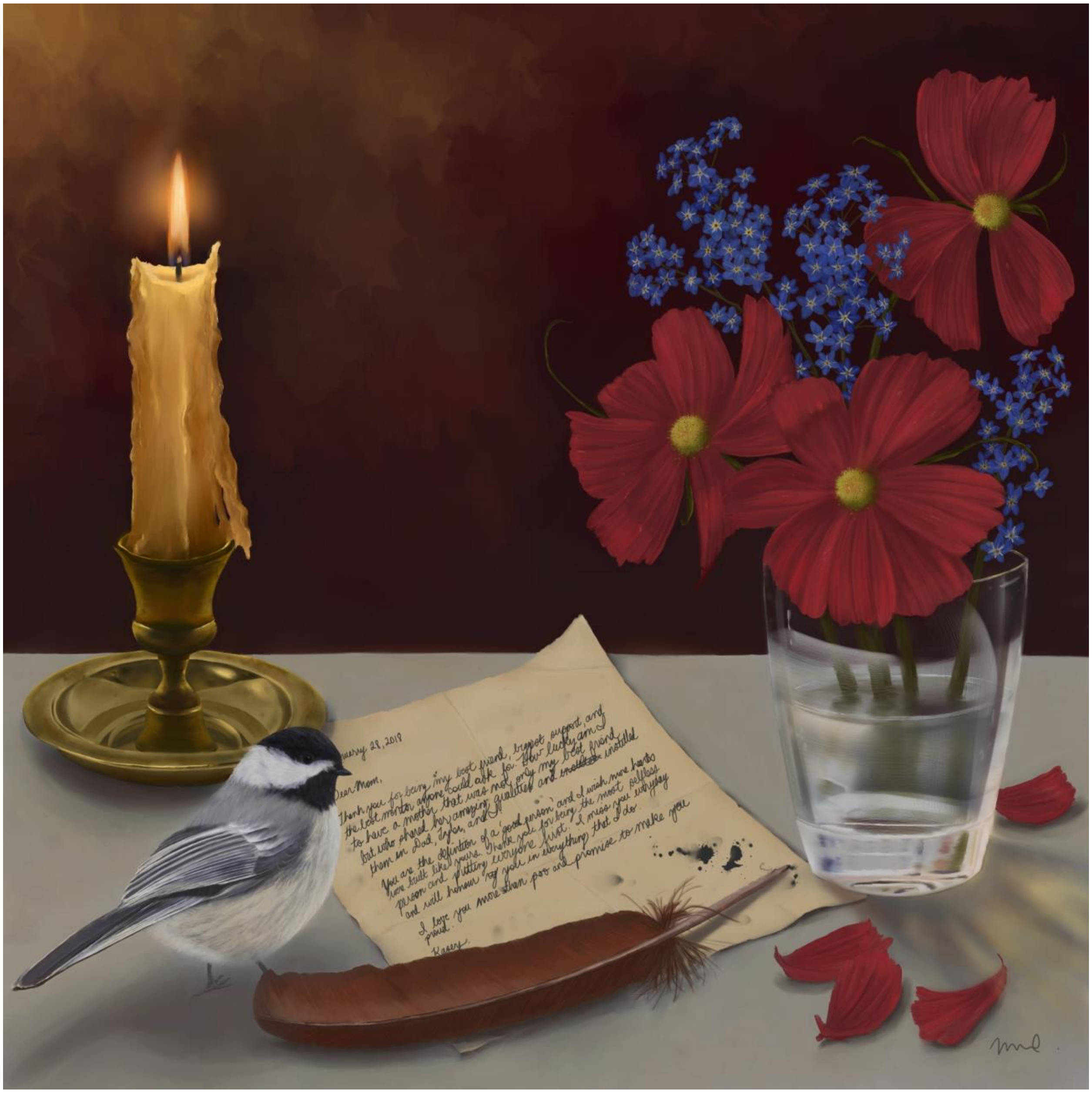

Painting 7: *Dear Michelle*

© 2020 Azad MA et al. *JAMA Network Open.*

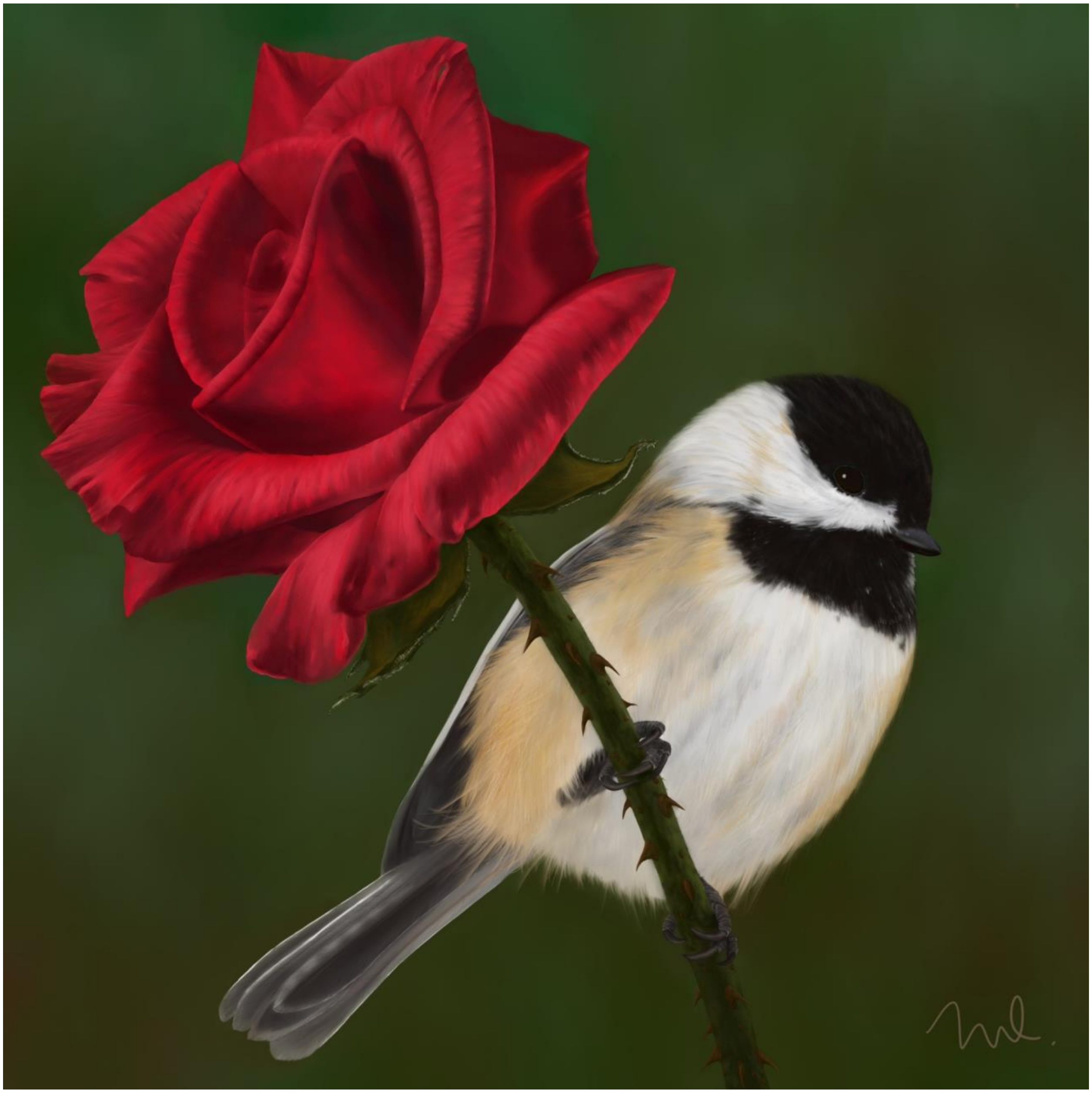

Painting 8: *For Rose*

© 2020 Azad MA et al. *JAMA Network Open.*

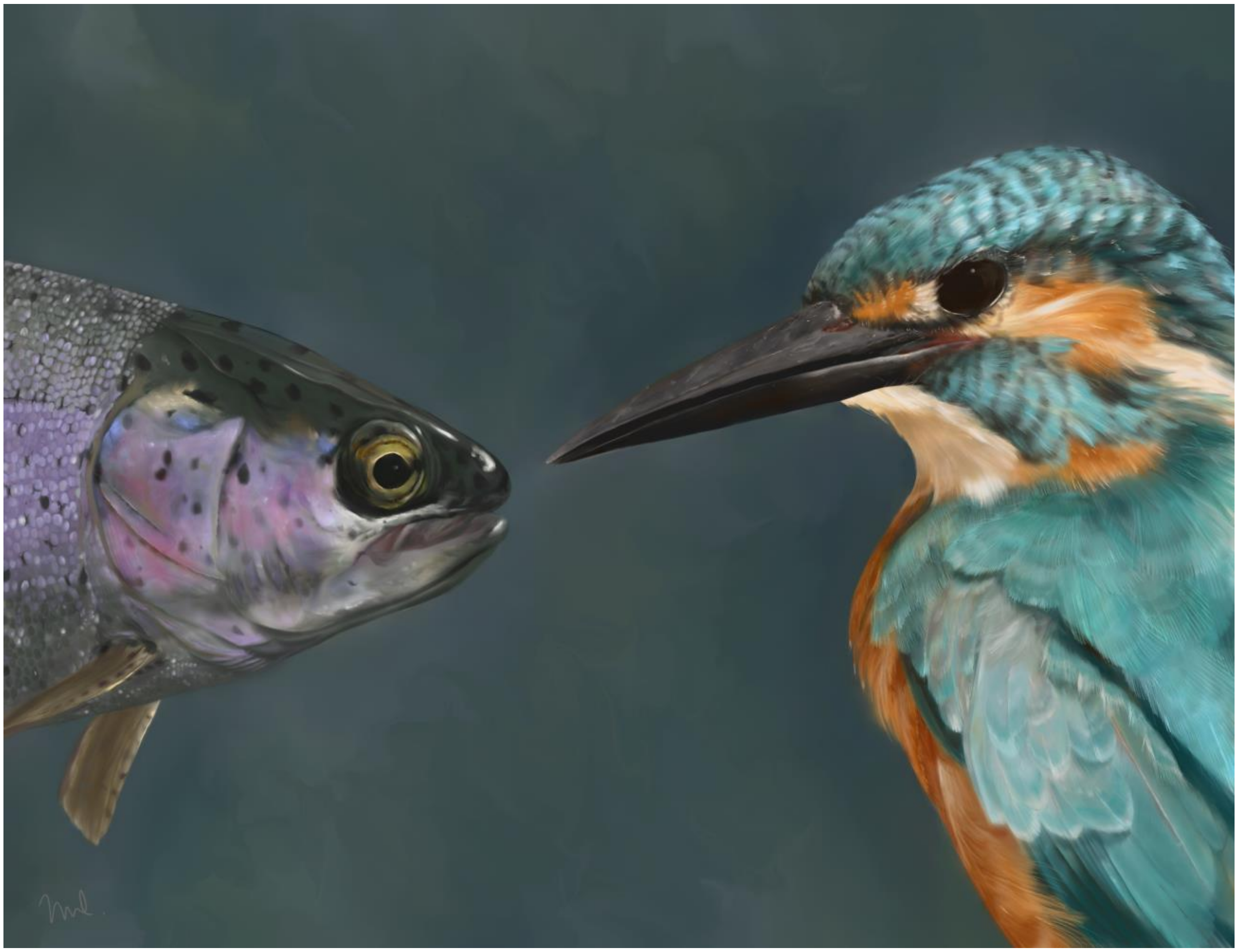
Painting 9: *The Fisherman*

© 2020 Azad MA et al. *JAMA Network Open.*

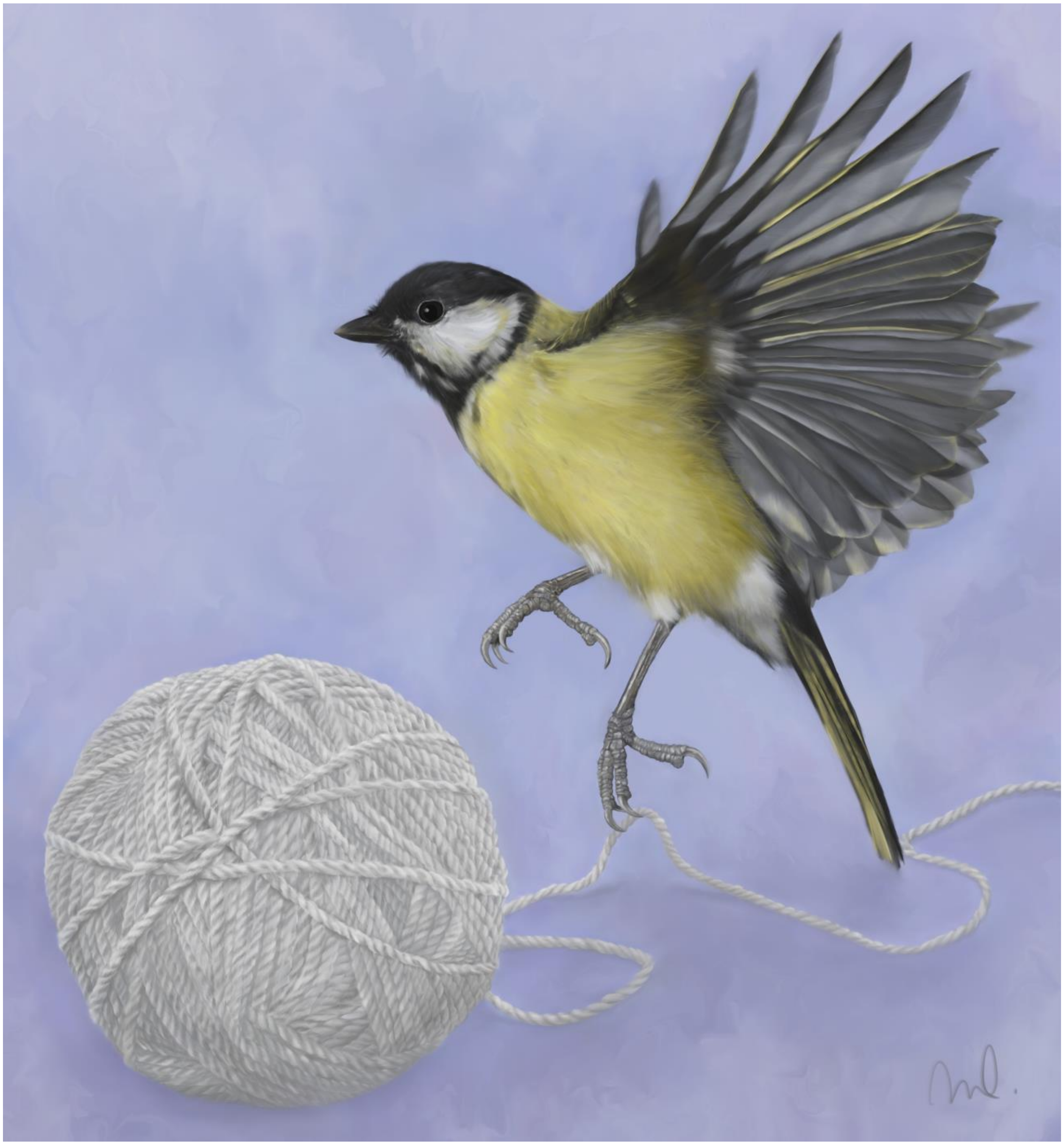

Painting 10: *Looking Back*

© 2020 Azad MA et al. *JAMA Network Open.*
